# First do no harm: uterine natural killer (NK) cells in assisted reproduction

**DOI:** 10.1093/humrep/dev098

**Published:** 2015-05-07

**Authors:** Ashley Moffett, Norman Shreeve

**Affiliations:** 1 Department ofPathology, University of Cambridge, Cambridge, UK; 2 Centre for Trophoblast Research, University of Cambridge, Cambridge, UK; 3 Department of Obstetrics & Gynecology, University of Cambridge, Cambridge, UK

**Keywords:** uterine natural killer cells, assisted reproduction, miscarriage, immunotherapy, embryo

## Abstract

Natural killer (NK) cells are a type of lymphocyte circulating in peripheral blood named because of their effector functions in killing target cells. Immune cells that share similar phenotypic characteristics but are poor killers populate the uterine lining at implantation and during early pregnancy when the placenta is established. The functions of these uterine NK (uNK) cells are essentially unknown but available data point to a role in regulating placentation in concert with other elements of the decidua and invading trophoblast cells. Despite the lack of scientific rationale and advice from clinical governing bodies, such as the Human Fertilisation and Embryology Authority, an increasing range of tests and therapies are still offered to women undergoing IVF or attending recurrent miscarriage clinics based on the myth that uterine NK cells need suppressing to prevent damage to the embryo. New treatments can be introduced at whim with subsequent demands for expensive trials to prove/disprove their efficacy. The evidence that targeting uNK or peripheral blood NK cells assists women with recurrent pregnancy failure is lacking. Healthcare professionals and patients should very carefully evaluate the practice of immunomodulation to enhance pregnancy outcome. A discussion on how to move towards stricter regulation of immunotherapy in non-hospital settings is now needed because it is clear that the potential risks and costs of these therapies outweigh any benefits.

## Introduction

Nearly 25 years ago three British scientists described a new type of immune cell in the human uterine mucosa (Bulmer *et al.*, 1991; King and Loke, 1991; Starkey *et al.*, 1991). Although these cells had previously been noted and given a variety of names (e.g. endometrial granulocytes, K cells), it was only around 1990 that they were identified as a type of lymphocyte belonging to the natural killer (NK) lineage. The cells were initially called uterine large granular lymphocytes based on their size and prominent cytoplasmic granules (King and Loke, 1991). When similar cells in the decidua of some rodents and primates were also found to be NK-lineage cells, the term ‘uterine NK cells’ (uNK) was introduced to describe these cells present during haemochorial placentation in a range of species (Croy *et al.*, 1996; King et al*.*, 1996).

NK cells circulating in blood were so called because they have the innate ability to kill some leukemic cell lines *in vitro* (Herberman *et al.*, 1975; Kiessling *et al.*, 1975). Retrospectively, uNK is a misnomer as they are poor at killing these same cell lines (King *et al.*, 1989). Regrettably, this name has been a driver for the myths that uNK can kill the embryo and are responsible for reproductive failure in women with recurrent miscarriage and failed IVF. The actual contribution of these maternal immune cells to success or failure in women with infertility and recurrent miscarriage is still unknown but there is no evidence that they kill trophoblast cells in these conditions (Moffett and Colucci, 2014). Despite this a large industry has grown up to treat women deemed to have excessively potent uterine ‘killers’. Apart from the lack of evidence base for these treatments, they are not without risk to young healthy women.

## Uterine NK cells

Granulated cells were identified over 100 years ago in the pregnant uterine mucosa and even described as a ‘type of leukocyte restricted to the decidua’ (Weill, 1921). It was not until the advent of immunohistochemistry that these cells were confidently identified as bone marrow-derived CD45+ leukocytes (Bulmer and Sunderland, 1983). There is general agreement that the dominant leukocytes present in the mucosa in the luteal phase and in early pregnancy are granulated lymphocytes, which are CD56+, but CD3− (so not T cells) and CD19− (not B cells). The CD56+ phenotype assigns them to the NK lineage, part of a wider group of lymphocytes known as innate lymphoid cells (ILCs) that all lack antigen specific receptors generated by somatic gene rearrangement (T and B cell receptors) and mediate homeostatic as well as developmental and defensive functions particularly at mucosal surfaces (Artis and Spits, 2015).

Peripheral blood NK cells (pbNK) are important as a first line of defence against viruses and are also important in controlling the early spread of tumours, functions mediated by both cytotoxicity and cytokine secretion. Unlike cytotoxic T cells, this killing can occur without prior stimulation (Karre, 2008). NK cells therefore play an important role in early innate immune responses to pathogens such as viruses (Horowitz *et al.*, 2012). In addition, NK cells drive adaptive immune responses to infections by influencing antigen-presenting cells (APC) (Vivier *et al.*, 2011). In peripheral blood, there are two major types of NK cells; 90% are CD56^dim^, CD16^+^ and 10% are CD56^bright^, CD16^−^ (Caligiuri, 2008). In contrast, uNK cells are CD56^superbright^, CD16^−^ and differ radically from pbNK in other phenotype markers and functional assays (King *et al.*, 1991; Starkey, 1991; Koopman *et al.*, 2003). The classical assay to assess pbNK function is killing of target cells that lack major histocompatibility complex class I molecules (known as HLA in humans) because this rules out any killing from circulating CD8^+^ cytotoxic T cells that are triggered to kill on recognition of HLA class I molecules. uNK killing *in vitro* of HLA-null target cell lines, such as K562, is very weak compared with pbNK (King *et al.*, 1989; Ferry *et al.*, 1990). Nevertheless, uNK can degranulate and this function can be used as an informative readout (Xiong *et al.*, 2013).

Where uNK originate from is still unclear. There might be *in utero* resident CD34^+^ stem cells (Keskin *et al.*, 2007; Vacca *et al.*, 2011) but it is more likely that uNK arise from immature circulating progenitors that migrate into the uterine mucosa and develop there (Male *et al.*, 2010). Whatever their origin, their unique properties are acquired *in utero* as CD56^+^ cells do proliferate and differentiate in the specialized progesterone-dominated microenvironment of the secretory endometrium and early decidua (Pace *et al.*, 1989).

uNK cells are the dominant immune cells in the uterine mucosa and account for >30% of cells in the stroma in the late secretory endometrium in humans (Bulmer and Lash, 2005). During pregnancy uNK are abundant throughout decidua but amass particularly at the site of placentation in close physical proximity to infiltrating trophoblast cells (Xiong *et al.*, 2013). They are also particularly prominent around spiral arteries and under the basement membrane of endometrial glands. Although there are some uNK cells still present at term they are really abundant early in gestation when the placenta is established. This is the time of rapid placental growth before the accelerated period of fetal growth that occurs in the second half of gestation (Wolf *et al.*, 1989).

NK cells are present in a range of tissues besides the uterus including the lymph nodes, thymus, spleen and liver (Yokoyama *et al.*, 2013). Whilst other tissue NK cells share similarities with uNK cells in that they are CD56^bright^, possess poor cytotoxic ability and are functionally different to pbNK, they differ from uNK cells as they do not possess the same repertoire of cell-surface receptors and their cellular subsets are regulated by different transcription factors than observed in uNK cells (Tayade *et al.*, 2005).

## Function of uNK cells

We can conclude from a range of data that there is a population of NK cells present in the uterine mucosa at implantation and in early pregnancy that is phenotypically and functionally quite dissimilar from pbNK and all other known ILC and NK subsets, including tissue resident NK cells. But there are still many unknowns, especially the roles of these cells in normal and abnormal pregnancy. The prevailing view, supported by their temporal and spatial association with the invading extravillous trophoblast (EVT), is that uNK cells play an important role in building a healthy placenta. A range of angiogenic factors secreted by uNK during decidualisation may contribute to the modification of the structure of pre-existing arteries, particularly the smooth muscle media (Hanna *et al.*, 2006). uNK cells may also play a role in decidualization itself since in mice lacking NK cells the decidua is abnormal and fetal weights are reduced (Ashkar *et al.*, 2000; Barber and Pollard, 2003).

Both genetic and other circumstantial evidence suggests that uNK contribute to the regulation of placentation by the decidua, the site where a territorial boundary is drawn between the mother (decidua) and her baby (trophoblast) (Moffett and Colucci, 2014). It is important to stress that exactly how they do this is still a mystery. When the spiral arteries are invaded by EVT complete destruction of the media is seen, an essential modification to increase blood flow to the fetus. Many studies suggest uNK can alter the depth and pattern of trophoblast invasion, best shown in a rat model (Chakraborty *et al.*, 2011). From the limited good quality evidence available, by acting either directly on the arteries and/or on invading trophoblast, uNK maintain the balance between excessive trophoblast intrusion and defective placentation; the latter increases the risk of miscarriage and pre-eclampsia and other disorders known collectively as the Great Obstetric Syndromes (Brosens *et al.*, 2011).

Evidence for the need to activate uNK comes from genetic studies in pre-eclampsia where variants of NK receptor genes, killer immunoglobulin-like receptors (*KIR*), have been analysed in case–control cohorts. *KIR* are highly polymorphic, expressed by uNK and bind and respond to fetal HLA-C molecules present on EVT (Sharkey *et al.*, 2008). The *HLA-C* locus is also polymorphic and this extreme variability in both maternal *KIR* and fetal HLA-C ligands means that each pregnancy is likely to have different combinations, even from the same parents (Moffett *et al.*, 2015). Women with a set of *KIR* genes that confer highly inhibitory signals to uNK are at risk of pre-eclampsia when the fetus inherits a paternal *HLA-C* allele that carries a C2 epitope. Women are protected if they happen to possess *KIR2DS1*, an activating KIR, which also binds this C2 epitope (Hiby *et al.*, 2004, 2010). Our own studies supported by *in vitro* experiments thus indicate that a certain degree of uNK cell activation promotes trophoblast invasion (Xiong *et al.*, 2013). Mouse studies also show that strong inhibition of uNK impedes both uterine vascular remodelling and fetal growth (Kieckbusch *et al.*, 2014). This is all in keeping with the complete lack of experimental evidence for the view that uNK need ‘suppressing’.

## NK testing

Despite this, soon after uNK were described, the notion that killing of the embryo by uNK was responsible for miscarriages and IVF failure was mooted. A clinic in Chicago, IL, USA, introduced blood tests to measure numbers of pbNK and their levels of killing in women with reproductive failure (Chao *et al.*, 1995). Any woman deemed to have ‘high’ levels was then offered a range of therapies (Ruiz *et al.*, 1996). These treatments have changed over the years but are intended to suppress NK cells from attacking the embryo or to have other ill-defined effects on the maternal immune system.

Quotes from a number of websites are revealing: ‘If the women's immune system for any reason identifies the embryo as foreign, only one thing will happen—she will begin to fight it’ (Ndukwe, 2015); ‘we found that some women's natural killer cells are so aggressive they attack the pregnancy, thinking the foetus is a foreign body’ (Shehata, 2014); ‘while these cells differ from those found in the blood, it is fortuitous that we can measure the natural killer activity of the cells in the blood and learn much about the activity of those within the uterus’; ‘there are couples who produce embryos that are misinterpreted by the immune system as foreign objects or even cancer cells. These embryos are repudiated and each attempt at pregnancy makes the problem worse until the uterus behaves like a “den of lions” and every pregnancy attempt fails’ (Alan E. Beer Centre ©, 2015).

These emotive claims lack any supporting evidence. uNK cells are never in contact with the embryo; they are only in contact with placental trophoblast cells. They cannot kill trophoblast unless exposed to high doses of interleukin (IL)-2, a cytokine absent from the normal pregnant uterus (Jokhi *et al.*, 1994; Lim *et al.*, 1998). Furthermore, uNK cells are quite distinct from pbNK cells and any parameter measured from a blood test cannot reflect their functional capabilities. This is unsurprising since the expression of activating and inhibitory receptors that regulate NK cell function differs considerably between pbNK and uNK (Moffett-King, 2002; Sharkey *et al.*, 2008).

The normal range for NK cell numbers and activity in peripheral blood from normal individuals varies widely, at ∼5–30% of mononuclear cells (Bisset *et al.*, 2004; Sacks *et al.*, 2012). Notably, however, pbNK are never measured routinely in any clinical condition apart from haematological disorders, such as leukaemia, because no clinically useful information is obtained. Indeed, because of the absence of any routine tests for pbNK in most hospital laboratories, blood samples taken in fertility clinics in the UK are often sent abroad for a range of expensive ‘immune’ tests including equally questionable ‘Th1/Th2 ratios’. Although there is no rationale for measuring any parameters in pbNK, this ‘NK testing’ has now spread across the world and is commonplace in a number of well-known fertility clinics.

In 2008 advice from the Royal College of Obstetricians and Gynaecologists (Rai, 2008) echoed by the Human Fertilisation and Embryology Association (HFEA) stated that ‘measurement of peripheral blood NK cell numbers or activity as a surrogate marker of events at the maternal-fetal interface is inappropriate’ and is ‘no better than tossing a coin’ (Rai *et al.*, 2005; HFEA, 2010). Despite this and the current paucity of strong evidence to suggest otherwise, it is interesting to speculate why this practice continues unabated today. One obvious reason is the pervasive and accessible idea that suppression of the immune system has a role in pregnancy success, although this is based on old ideas not relevant to NK cells.

T cell suppression is certainly needed for successful transplantation and it was Peter Medawar himself who introduced the concepts of the fetal transplant and maternal immunosuppression (Medawar, 1953). The possible need for T cell tolerance to accommodate the fetus has been extensively researched for decades (Mellor and Munn, 2000). Despite this, 60 years on there is still no experimental evidence to support the notion that systemic maternal tolerance of T cells is required for successful human pregnancy (Colucci *et al.*, 2014), although elegant mouse experiments have provided evidence that lack of regulatory T cells does lead to fetal demise (Rowe *et al.*, 2012; Samstein *et al.*, 2012). One reason for the apparent inconsistency with the mouse studies is the difficulty in distinguishing between systemic T cell responses to fetal somatic cells crossing into the maternal circulation from those local uterine T cell responses to invading EVT (Moffett and Colucci, 2014; Nancy and Erlebacher, 2014). T cells are normally relatively sparse at the site of placentation and have not been shown to damage the implanting placenta in humans. In addition, B cells or plasma cells are absent in decidua, which suggests that antibodies to invasive EVT are normally never generated. Thus, there is no convincing evidence that IVF failure or recurrent pregnancy loss result from adverse adaptive T or B cell immune responses to the placenta.

## Measurement of uNK cells

More recently attempts have been made to correlate numbers of uNK cells during the luteal phase with successful implantation/placentation (Tuckerman *et al.*, 2007; Kuroda *et al.*, 2013). A major problem in counting uNK in endometrium is their huge variability over the menstrual cycle, with a rapid surge in numbers after ovulation due to proliferation triggered by progesterone-mediated induction of IL-15 in stromal cells (Wilkens *et al.*, 2013). In addition, the presence of oedema, depth from the surface epithelium and site in the uterus can all affect uNK numbers. Even without these problems there is no reason to correlate *numbers* of uNK present prior to pregnancy with any putative harmful or beneficial function. As has been pointed out by others, because uNK numbers do change so dramatically over the luteal phase, any alteration from normal control women (women of reproductive age with no known infertility) may just reflect maturational delay or acceleration affecting the whole mucosa (Russell *et al.*, 2013). In other words, any deviations in the hypothalamic/pituitary/ovarian (HPO) axis will affect all components of the cycling mucosa including uNK proliferation (Kosova *et al.*, 2015). Pre-conceptual elevated numbers of uNK can be reduced by prednisolone in women with recurrent miscarriage (Quenby *et al.*, 2005) but this correlation does not account for the wider effects of steroid therapy on the HPO axis and the global effect of steroids on lymphocytes. Given the invasive procedure necessary and the difficulty in standardizing this test between different labs and operators, counting uNK in endometrium should only be considered in research settings. All these tests are expensive; even in clinics where customers are warned that *‘*there is no absolute evidence that uterine NK cells are destructive and attack placental or embryonic cells’ (Natural Killer Cell Testing, 2015) couples can expect to pay >£1000 for a combined uNK and pbNK test prior to undergoing an assisted reproduction cycle.

## Range of ‘immune’ treatments

In the 1980s women were given infusions of paternal leukocytes for treatment of infertility and recurrent miscarriage, now known as lymphocyte immunotherapy (LIT). The rationale and mechanism for this was always confused but was supposed to stimulate a ‘beneficial’ immune response to paternal antigens (Mowbray *et al.*, 1985). This treatment was subsequently banned in 2002 by the US Food and Drug Administration (FDA), who wrote to all physicians believed to be using LIT as a therapy *‘*that the injectable products used do not have the required FDA approval and are considered investigational drugs that pose several safety concerns’ (Wong *et al.*, 2014). The reasoning behind LIT was always unclear because the *systemic* maternal immune system is normally stimulated by paternal antigens expressed by the fetus, as illustrated by the generation of maternal antibodies specific to paternal HLA allotypes and blood group antigens (e.g. Rhesus D). Importantly, this needs to be considered quite separately from *uterine* immune responses to EVT that occur during placentation.

Over the subsequent years a wide range of other therapies has been introduced seemingly at random: these include tumour necrosis factor alpha (TNFα) inhibitors, intravenous immunoglobulins (IVIg), Intralipid, prednisolone or dexamethasone and granulocyte-colony stimulating factor. Table I summarizes the indications for the normal use of these therapies, their side effects and costs. Some of these therapies will certainly affect the maternal immune system but not necessarily in a beneficial way. The risk of infection, especially tuberculosis, is a side effect of TNFα inhibitors, steroids are globally immunosuppressive and IVIg (not recommended for use in IVF failure or recurrent pregnancy loss by the UK Dept. of Health) can blockade Fc receptors on other immune cells, alter cytokine production and neutralize complement components, thereby possibly interfering with maternal immune defence mechanisms to pathogens. A salutary report describes a case of systemic candidiasis presenting at 18 weeks gestation resulting in fetal loss following administration of TNFα inhibitor, prednisolone and Intralipid during assisted reproduction (Akhanoba, 2014). Around 40% of IVIg users will experience mild adverse events, and in serious cases can suffer from anaphylaxis (Berger, 2013). An additional ethical concern is the diversion of IVIg from patients with serious conditions necessitating strict allocation of the limited supplies available (Department of Health, 2011). Whether or how any of these therapies might affect uNK function is unknown and hard to predict, given we are still so uncertain of the exact role of uNK in successful or failing pregnancy.

**Table I:** A summary of agents used for immunomodulation in assisted reproductive technology.

Drug	Cost	Common clinical uses	Some known side effects or adverse events
Lipid Emulsion e.g. *Intralipid* ©	Approx. £300 per infusion in clinic setting	Parenteral nutrition, administered with propofol, cardio-protection in bupivacaine toxicity (Picard, 2006)	Hepatomegaly, jaundice, cholestasis, splenomegaly, thrombocytopenia, leukopenia and fat overload syndrome (<1% occurrence in clinical trials) (FDA, 2007)
Intravenous Immunoglobulin (IVIG)	Approx. £1500 per infusion (may vary depending on dose) in clinic setting	Primary and secondary antibody deficiency states, haematological disorders, neurological conditions, other uses, e.g. solid organ transplantation (DoH, 2011)	Aseptic meningitis, renal failure, thromboembolism, haemolytic reactions, anaphylactic reactions, lung disease, enteritis, dermatologic disorders and infectious diseases (Stiehm, 2013)
Corticosteroids	Net price 28-tablet pack £1.86 (BNF, 2015)	Suppression of inflammatory/allergic disorders, inflammatory bowel disease, asthma, croup, rheumatic disease, eye and ear conditions (BNF, 2015)	Gastric ulceration, Cushing's syndrome, diabetes, hypocalcaemia, osteoporosis, skin thinning, dry skin, high blood sugar (BNF, 2015)
Anti-tumour necrosis factor (Anti-TNF)	Net price £352.14/40 mg syringe (BNF, 2015)	Autoimmune disorders, e.g. rheumatoid arthritis, ankylosing spondylitis, inflammatory bowel disease, psoriasis (BNF, 2015)	Infection, lymphoma, demyelinating disease, autoantibody induction, congestive heart failure, injection site reactions, lupus-like syndrome (Scheinfeld, 2004)
Granulocyte-Colony Stimulating Factor (G-CSF)	Net price 600 mcg/ml £52.70, 0.5-ml prefilled syringe (BNF, 2015)	Neutropenia (various clinical types), severe or recurrent infections in advanced human immunodeficiency virus infection (BNF, 2015)	Mucositis, splenic enlargement, hepatomegaly, transient hypotension, epistaxis, urinary abnormalities, osteoporosis, exacerbation of rheumatoid arthritis, anaemia, pseudogout (BNF, 2015)

BNF, British National Formulary; DoH, Department of Health; FDA, Food and Drug Administration.

## Conclusions

A recent Cochrane review found ‘Paternal cell immunization, third-party donor leukocytes, trophoblast membranes, and intravenous immunoglobulin provide no significant beneficial effect over placebo in improving the live birth rate’ (Wong *et al.*, 2014). Introducing more therapies with no underpinning from basic science and then testing these with costly trials cannot be the way forward. The difficulties in studying trophoblast/decidua interactions events in normal and abnormal pregnancies in the first few weeks of pregnancy are considerable but this must be the focus of research by clinicians and at the bench.

Since writing a similar review 10 years ago, it is worth reflecting why there has been limited scientific progress yet even more therapies that offer no benefit and possible harm. Private IVF and miscarriage clinics can use these treatments at whim driven by commercial gain because, whilst HFEA approved licences are required to perform IVF, no such licences are required for reproductive immunology treatments. It is understandable that commercial success and patient demand for the continued development of such tests and treatments continues to drive clinical practice. Even the most informed patients are often willing to ‘try anything’ to achieve a live birth. The misrepresentation by the media of poorly designed clinical research related to uNK function—e.g. ‘My body tried kill my baby’ (Fricker, 2007) and ‘The killer cells that robbed me of four babies’ (Barber, 2011)—has only served to heighten this demand (Shreeve and Sadek, 2012). This can leave clinicians in potentially compromising situations where a lack of scientific evidence can be superseded by a patient's will to fall pregnant through the use of ‘safe’ immunomodulation.

We acknowledge that some clinicians do appear to ‘believe fervently’ in the merits of immunotherapy (Fishel, 2013). Nonetheless, it is surely no longer acceptable for licensed medical practitioners to continue to administer and profit from potentially unsafe and unproven treatments, based on belief and not scientific rationale. Allowing the regular administration of potentially harmful medicines to go virtually unchecked sets a dangerous precedent, irrespective of the assumed clinical benefit. In accordance with the major clinical governing bodies, healthcare professionals and patients should very carefully evaluate the practice of immunomodulation to enhance pregnancy outcome. The time has also come for a move towards stricter regulation of immunotherapy in non-hospital settings.

## Authors' roles

N.S. wrote the manuscript. A.M. provided the conceptual ideas, wrote the manuscript and approved of the final submitted version.

## Funding

A.M. acknowledges funding support from the Wellcome Trust and Centre for Trophoblast Research, University of Cambridge, UK. Funding to pay the Open Access publication charges for this article was provided by the University of Cambridge.

## Conflict of interest

None declared.
